# Effect of Hyaluronic Acid on Palatal Wound Healing—A Randomized Crossover Clinical Trial

**DOI:** 10.1111/clr.70159

**Published:** 2026-07-16

**Authors:** Madeleine Dahi, Sara Amash, Kristina Bertl, Florian Beck, Patrick Heimel, Andreas Stavropoulos

**Affiliations:** ^1^ Periodontology, Faculty of Odontology University of Malmö Malmö Sweden; ^2^ Department of Periodontology, Dental Clinic, Faculty of Medicine Sigmund Freud University Vienna Austria; ^3^ Department of Periodontology Blekinge Hospital Karlskrona Sweden; ^4^ Division of Oral Surgery, University Clinic of Dentistry Medical University of Vienna Vienna Austria; ^5^ Karl Donath Laboratory for Hard Tissue and Biomaterial Research, Division of Oral Surgery, University Clinic of Dentistry Medical University of Vienna Vienna Austria; ^6^ Austrian Cluster for Tissue Regeneration Vienna Austria; ^7^ Ludwig Boltzmann Institute for Traumatology, The Research Center in Cooperation With AUVA Vienna Austria; ^8^ Division of Conservative Dentistry and Periodontology, University Clinic of Dentistry Medical University of Vienna Vienna Austria; ^9^ Department of Periodontology, School of Dental Medicine University of Bern Bern Switzerland

**Keywords:** graft harvesting, hyaluronic acid, pain, palatal wound healing, randomized controlled clinical trial, re‐epithelization, soft tissue grafting

## Abstract

**Aim:**

To assess the effect of a hyaluronic acid (HyA) containing gel on patient‐related outcomes (PRO) and wound healing after palatal punch‐biopsy representing a free gingival graft.

**Material and Methods:**

A punch‐biopsy (6 mm diameter, 2 mm thickness) was harvested from one side of the palate at Day 0 and from the contralateral side at Day 21. Sites were randomly allocated to either 0.3% HyA containing gel (test) or sterile saline solution (control), professionally applied after harvesting and then self‐applied three times/day for 7 days. Up to 21 days PRO‐related questionnaires were answered and intraoral scans and photographs recorded to assess wound re‐epithelialization, tissue refill, and color match.

**Results:**

Eighteen of 25 recruited participants were analyzed. Pain perception, difficulties with eating/drinking, and taste alterations significantly decreased over time and were hardly experienced after Day 7 in both groups (*p* > 0.05). Participants reported significantly more often a positive experience after applying HyA compared to placebo, that is, in 8 versus 1 out of 18 cases. The residual wound area was significantly smaller in the test compared to control group at Day 7, and cases with a thicker palatal tissue showed tendency for faster re‐epithelialization.

**Conclusion:**

Despite the potential limitations of the present study (i.e., possibility of a carryover effect, questionable blinding of the participants), repeated local application of a 0.3% HyA containing gel appears to only accelerate re‐epithelialization in an open palatal wound and provide a positive experience after application, but it did not improve any PRO in such small‐sized wounds compared to sterile saline application.

**Trial Registration:**

ClinicalTrials.gov identifier: NCT05099718

## 1 Introduction

Autogenous soft tissue grafting is frequently performed around dental implants, natural teeth, and at edentulous crests; commonly an epithelialized free gingival graft (FGG) is used to widen the band of keratinized tissue and a connective tissue graft (CTG) to increase the thickness of the gingival/mucosal tissue (Zucchelli et al. 2010). Compared to soft tissue substitutes, using autogenous tissue is still considered the gold standard in both indications, that is, for dental implants as well as for natural teeth (Bertl, Melchard, et al. 2017; Thoma et al. 2018; Bertl et al. 2021; Montero et al. 2022; Valles et al. 2022; Rotundo et al. 2024). In this context, a CTG is nowadays often harvested like a FGG, intra‐ or extraorally de‐epithelialized (Zucchelli et al. 2010), which has the major disadvantage of leaving an open wound with secondary intention healing at the palatal donor site, and is considered to contribute to patient postoperative morbidity (Burkhardt et al. 2015) that can negatively affect patient compliance, confidence in the treatment, and willingness to undergo future treatment (Tavelli, Barootchi, Di Gianfilippo, et al. 2021). Hence, any technique or adjunct measure to reduce patient discomfort and morbidity are clinically relevant (Tavelli, Barootchi, Di Gianfilippo, et al. 2021; Silva et al. 2023). It has been previously shown that graft size including graft thickness as well as the thickness of the remaining tissue at the palate influence postoperative morbidity (Zucchelli et al. 2010, 2014; Tavelli et al. 2018; Wyrębek et al. 2018).

As it is difficult to change the required graft size and the thickness at the palatal donor site, various adjuncts/interventions are frequently tested for promoting palatal wound healing and thereby reducing postoperative morbidity. Three recent reviews (Silva et al. 2023; de Almeida et al. 2024; Jankowski et al. 2024) tried to summarize the currently available options, ranging from biologics to blood‐derived products, customized palatal stents, local dressings, hemostatic agents, laser therapy, etc. While some measures appear to reduce pain/discomfort, others improve re‐epithelialization and wound healing, and/or reduce postoperative bleeding events. However, none of them has been proven clearly superior and there is no consensus on the best option; in addition, many of the original studies show relatively low methodological quality and relevant risks of bias (Silva et al. 2023; de Almeida et al. 2024; Jankowski et al. 2024), which in turn urges for further research on this topic.

In this context, hyaluronic acid (HyA), a biologic, is suggested to support/promote soft and hard tissue as well as periodontal healing (Bertl et al. 2015; Pilloni et al. 2025; Bayyigit et al. 2026). HyA is a naturally occurring glycosaminoglycan, which appears to possess antibacterial, anti‐inflammatory, pro‐angiogenic, and wound healing promoting properties (Dahiya and Kamal 2013; Bertl et al. 2015; Kobayashi et al. 2020; Pilloni et al. 2025; Bayyigit et al. 2026). HyA has indeed been tested as adjunct to promote palatal wound healing after harvesting autogenous soft tissue grafts. A recent systematic review (Joshi et al. 2024) summarized the effect of HyA in this indication, showing overall promising, positive results regarding wound healing and patient‐reported outcomes (PRO). Based on these findings, the present study examined the potential of a specific commercially available HyA product for in terms of palatal wound healing. More specifically, the aim of this study was to assess the effect of repeated local application of a 0.3% HyA containing gel on PRO and wound healing with pain as the primary outcome parameter after palatal punch‐biopsy representing a FGG.

## 2 Methods

### 2.1 Study Design and Protocol

The present study is a randomized controlled clinical, crossover, examiner‐blinded trial and is reported in accordance with the standards of reporting randomized crossover trials (CONSORT Statement) (Dwan et al. 2019). The study protocol was approved by the regional ethical review board (Dnr. 2020‐01018) and registered on ClinicalTrials.gov.

### 2.2 Sample Size Calculation

The sample size calculation is based on pain perception and two publications, that is, on previously published data specifically on palatal wound healing (Yıldırım et al. 2018) as well as on a review on minimum clinically important difference in acute pain (Olsen et al. 2017); for allowing direct comparison the VAS values of Yildrim et al. were transformed from a scale 0–10 to 0–100. A mean difference of 25 mm with a standard deviation of 30 mm, on a VAS, was considered as relevant. Hence, based on 90% power, an alpha error of 0.05, and the crossover design a minimum of 16 participants was required (Chow and Wang 2007). For more details see the Supporting Information.

### 2.3 Study Population and Recruitment

Volunteers were recruited at the Faculty of Odontology (Malmö University, Sweden) between 31 October 2021 and 19 September 2022. The following eligibility criteria were applied: (1) age between 18 and 65 years, (2) systemically healthy and no conditions or medication intake potentially interfering with healing after a soft tissue biopsy, (3) healthy periodontal conditions (Chapple et al. 2018), (4) no known sensitivity to any of the ingredients of the test or control product, (5) not pregnant or breastfeeding, and (6) not heavy smoking (i.e., no regular tobacco consumption > 10 cigarettes/day). All volunteers received information on the study purpose and associated risks and provided written informed consent prior to active participation; as a minor compensation they received a cinema ticket.

### 2.4 Test and Placebo/Control Product

According to the crossover design of the present study, all volunteers were treated with both the test and placebo/control product. The test product was a gel containing HyA (0.3%, non‐crosslinked, middle molecular weight, nonanimal origin; GUM Aftaclear Gel; Sunstar Europe SA, Etoy, Switzerland), while the control product was physiological saline solution. The test product is a CE‐marked and FDA‐approved medical device Class IIa for the local treatment of mouth ulcers (e.g., aphthae) and small lesions in the mouth.

### 2.5 Randomization

A computer‐assisted randomization table was used to randomly assign the side of the palate and the treatment group for the first biopsy site. The second biopsy site was then on the other side of the palate and was allocated to the other group.

### 2.6 Splint Preparation and Standardization of the Biopsy Harvesting

At the first appointment a maxillary alginate impression was taken and a gypsum cast produced. An ESSIX guide of 1 mm thickness was vacuumed on the cast to manufacture an individualized splint with two symmetrical round openings (6 mm in diameter) between the first premolar and first molar in a distance of 5 mm to the gingival margin (Figure 1). This splint was used to standardize the biopsy harvesting by means of a biopsy punch‐blade (6 mm in diameter). Each biopsy punch‐blade was additionally equipped with a 3D printed plastic stop ensuring that the biopsy punch‐blade was intruding exactly 2 mm into the palatal tissue, providing a depth indication for the apical cut, which was performed manually with a surgical blade. Altogether, the intended size of the harvested circular biopsy was 6 mm in diameter and 2 mm thick (Figure 1).

**FIGURE 1:**
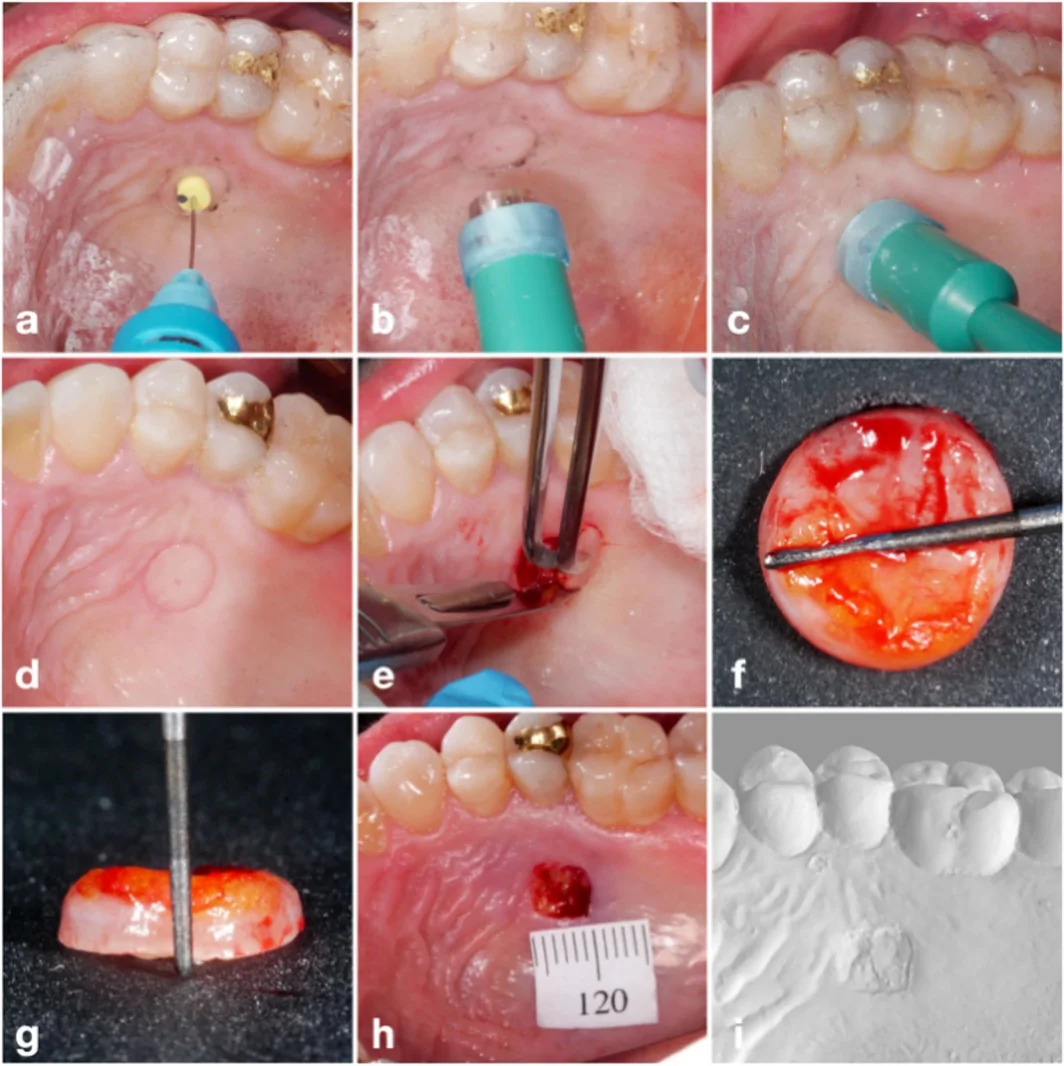
Overview of the procedure; (a) measuring of the palatal thickness; (b–e) harvesting of a standardized biopsy with a punch knife with a stopper ring controlling insertion depth and a 15C surgical blade; (f, g) the biopsy was 6 mm in diameter and 2 mm thick; (h, i) recording of the site with intraoral photographs, including a paper ruler for calibration, and intraoral scans.

### 2.7 Biopsy Harvesting and Parameters Assessed Prior to‐ and at Harvesting

First, the upper jaw including the palate was scanned with an intraoral scanner (Omnicam intraoral scanner; Dentsply Sirona, Charlotte, North Carolina, USA) and the intended harvesting site was photographed with a digital camera (DMC‐G3; Panasonic, Osaka, Japan) with a macro lens (M.Zuiko Digital ED 60 mm F2.8 Macro; Olympus, Tokyo Japan) and a ring flash (Metz Mecablitz 15 MS‐1 Macro Ringlight, Zirndorf, Germany) using an intraoral mirror to achieve an approximately 90° angle to the surface of the palate. Thereafter, the splint was inserted and the palatal thickness at the center of the harvesting site was assessed using a needle mounted with a silicone stop that was inserted perpendicularly until touching the bone. The needle insertion depth was secured by placing the silicone stop in direct contact with the palatal mucosa. Then the needle was retracted for about 2 mm and local anesthesia (2% Xylocaine Dental; 2% Lidocaine HCI and Epinephrine, Dentsply, Charlotte, North Carolina, USA) was injected; after removing the needle, the needle with the silicone stop juxtaposed to a paper ruler was photographed. The distance from the tip of the needle to the base of the silicon stope (i.e., the insertion depth) was then measured in an image analysis program (GIMP, version 2.10, Charlotte, North Carolina, USA), after calibrating the program using the paper ruler included in the photograph. Thereafter, the above‐described biopsy punch‐blade with the plastic stop was inserted into the palatal tissue and the biopsy harvested at the predefined position in a standardized fashion, using a 15C surgical blade, resulting in a circular 6 mm and ca. 2 mm thick FGG (Figure 1). All biopsies were harvested by the same experienced periodontist (K.B.), blinded to group allocation, that is, group allocation was kept concealed until biopsy harvesting was completed. After the harvesting, either the test or control product (randomly assigned) was applied on a gauze, which was then used for compressing the wound to achieve hemostasis. After hemostasis was achieved the intraoral scan and photograph (including a paper ruler) were repeated in the same way as described above (Figure 1). The participants received verbal and written instructions to (1) consume painkillers as needed (Paracetamol 500 mg, max. 2 tablets × 4 times per day), (2) use additional gauzes if any postoperative bleeding occurred, (3) use Q‐tips to apply the allocated test or control product to the harvesting site three times per day for 7 days, that is, the participants were neither informed about treatment allocation nor about the consistency of the test product (i.e., gel or fluid), (4) brush carefully at the palatal aspect, (5) abstain from using mouthwashes and any night guard covering the palate, and (6) to fill a form for the following parameters on Days 1 and 2:

- Number of painkillers used
- Frequency of postoperative bleeding
- Pain perception (visual analogue scale [VAS] 10 cm)
- Difficulties while eating/drinking (VAS 10 cm)
- Limitations regarding food consumption (yes/no)
- Experience of any taste alteration (VAS 10 cm)

### 2.8 Parameters Assessed During Follow‐Up

Patients were examined after 3, 7, 14, and 21 days by one blinded examiner (M.D. or S.A.) (Figure 2). At each examination, a maxillary intraoral scan and a photograph of the harvesting site were taken as described above. Further, a questionnaire containing the same parameters as for Days 1 and 2 was filled in. In addition, on Day 7, the participants were asked if they had a positive experience when applying the product (yes/no). Based on the intraoral scans and photographs, the following parameters were collected and assessed by a blinded examiner:

1. *Area*: The photograph of Day 7 was calibrated in Photoshop (Adobe Inc., San Jose, California, USA) and the non‐epithelialized wound area was marked with a selection tool and expressed as mm^2^.
2. *Volumetric assessment of the defect after biopsy harvesting*: The intraoral scans were saved as STL files and imported in the software GOM Inspect Pro (GOM Software 2022; Carl Zeiss GOM Metrology GmbH, Braunschweig, Germany). Artifacts or overhangs present on the surface scans were manually rectified. Further, the intraoral scan before biopsy harvesting (reference) was specified as CAD model and consecutive scans (Days 0, 7, 14, and 21) were initially aligned by a best‐fit algorithm to the CAD. Specifically, a local best‐fit alignment was performed by excluding the area of the harvesting site and those palatal aspects swollen due to the local anesthesia, that is, the alignment was restricted to the teeth and minor parts of the palate. The area of the harvesting site (ROI) was traced on the CAD model corresponding to the visible defect on scan of Day 0. A staged model ensured that this ROI was provided on all scans. At the ROI, a surface comparison was conducted between the reference scan (CAD model) and the consecutive scans (Days 7, 14, and 21) to assess the volume (volume deficiency and maximum defect depth) at the different time points. In particular, the inscribed volume between the surfaces was calculated by the integrated distance and the defect depth corresponded to the maximum deviation of the surface comparison within the ROI (Figure 3).
3. *Color match of the harvesting site compared to the surrounding tissue*: Segmentation and measurement of the color match based on the photographs was performed with Fiji (Schindelin et al. 2012). For each volunteer, photographs were combined into a single stack. On each slice of the stack, 4–8 landmarks, such as folds at the palatal tissue and gaps between the teeth, were marked using the multi point tool and added to the ROI manager. Based on the drawn landmarks, all time points were registered to the first time point using the “moving least squares” plugin (Schaefer et al. 2006). On the registered stack, three additional ROI were drawn on each image. The defect was drawn using the oval, lasso and polygon selection tools on Day 0 and copied to all other time points. Due to stretching or contraction of the palatal tissue, the exact position of the defect could move over time. In such cases, the defect ROI was moved to be centered on the new position of the defect, but the size of the defect ROI was the same at all time points. Secondly, a valid measurement area was drawn on each time point. This was a polygonal ROI which included all visible parts of the palatal tissue but excluded teeth and the ruler. Thirdly, on each time point a 5 mm line was drawn on the ruler for calibration of the scale. To normalize the colors between time points, color balancing was performed. On each time point, a balancing ROI was created from the manually drawn ROI. The defect ROI was subtracted from the valid measurement area ROI, creating an ROI containing just the palatal mucosa without the defect. In this ROI, the average intensity of each color was measured. Each color layer was then scaled so that the mean color was the same at all time points. As the palatal tissue was moist, it led to reflections of the light on the photograph. These reflections appear much brighter, up to a white color, than the surrounding tissue. To prevent these reflections from altering the color measurement, they were removed using a top hat filter with radius 10 without background subtraction. This filter is equivalent to a grayscale open filter. In fact, intensity peaks with a radius of < 10 pixels were removed and replaced with the intensity which surrounds the peak, removing the reflections. The color intensity was measured in two regions. The defect itself based on the manually drawn defect ROI and the surrounding palatal tissue as a 2 mm wide band around the defect with a 1 mm wide gap between the defect and the band. Based on the valid measurement area ROI, areas of the band which were not on the palatal tissue were excluded from measurement. The average intensity of each color layer in the defect was divided by the average intensity in the band. The output of the measurement was this ratio of the average for green and blue color layers for each time point. For statistical analysis the values from Days 3, 7, 14, and 21 were used.
4. *Visual judgment of epithelialization and wound visibility (yes/no)*: All photographs from prior to the biopsy and from Days 7, 14, and 21 were cropped, randomized, masked, and judged by four examiners (M.D., S.A., K.B., and A.S.). Each harvesting site was classified as (1) completely (yes) or not completely (no) epithelialized, and (2) visible (yes) or not visible (no). In case of disagreement, the case was discussed within the group and a conclusion taken.

**FIGURE 2:**
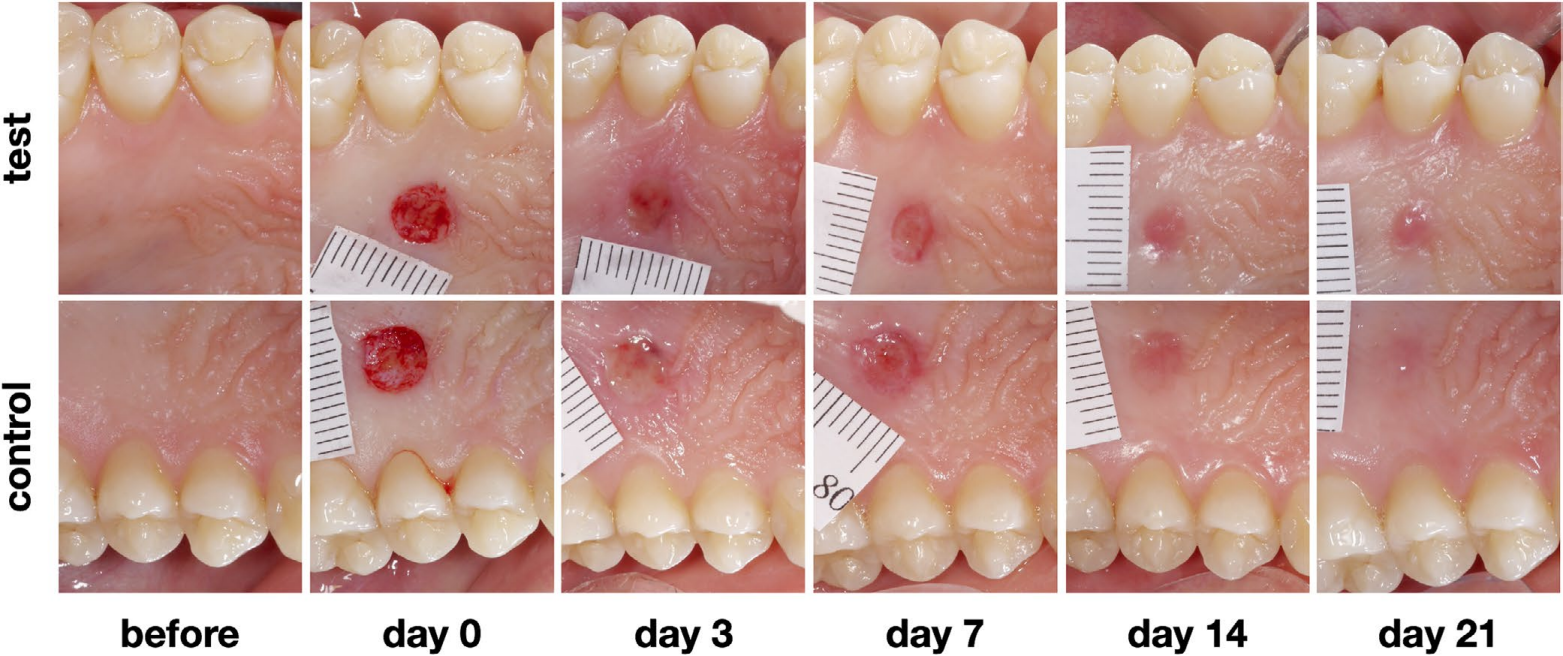
Representative patient case including all follow‐ups until Day 21. The upper line displays the site treated with the test product, while the lower line was treated with placebo.

**FIGURE 3:**
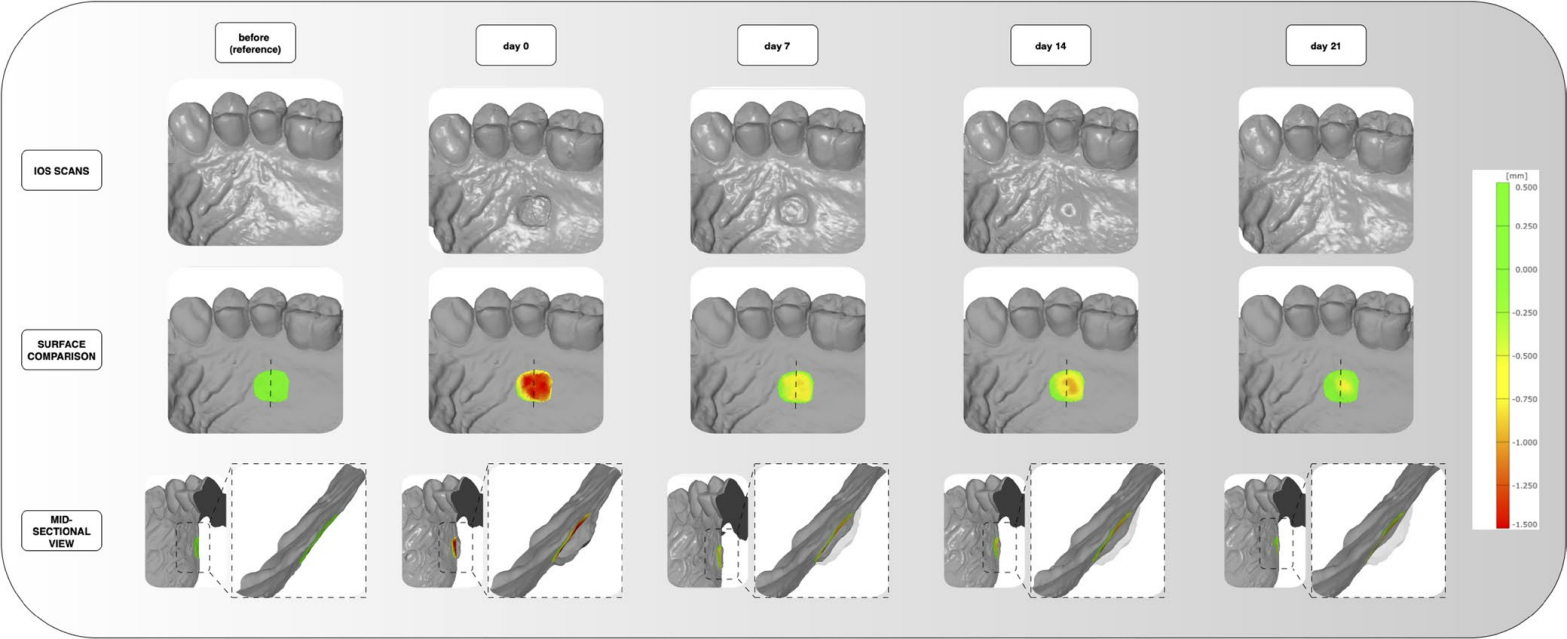
Assessment of the volume changes after alignment of the various intraoral scans at the different time points. Note, for illustration purposes the surface comparison (second row) is depicted as a colored distance map on the reference scan (CAD model). The scale was restricted to min/max values comprising surface deviations of all time points.

The same procedures and calculations were repeated for the contralateral site treated with the other product.

### 2.9 Statistical Analysis

All assessed parameters were reported descriptively, and their distribution was tested with the Shapiro–Wilk test. The palatal thickness (i.e., continuous) at the harvesting site receiving either the test or control product was compared using the paired *t*‐test, while the positive experience (i.e., categorical) after applying the test or control product was compared using the Exact McNemar test. The dropouts were compared to the finally analyzed population regarding gender distribution and group allocation (i.e., both categorical) using Fishers exact test and regarding age (i.e., continuous) using Mann–Whitney *U* test. Pain perception (VAS 10 cm) was defined as the primary outcome parameter, while the other outcome parameters were considered as secondary. Multilevel mixed‐effects linear regression analyses were used for the following outcome parameters (dependent variables): Pain perception (VAS 10 cm), pain killer consumption (total amount), difficulties with eating/drinking (VAS 10 cm), taste alterations (VAS 10 cm), bleeding events (total number), area at Day 7 (mm^2^), volume deficiency (mm^3^), maximum defect depth (mm), and color matching (%). Group allocation was defined as the main predictor (independent variable) and gender, age, and palatal thickness were included as a priori confounders, that is, all were included in the analysis per protocol. The random‐effects structure was set up with palatal side nested in patient. Further, if available, the time points and an interaction between time points and group allocation were included as confounders; whether the interaction was kept in the final model was determined with the Likelihood Ratio Test. A post hoc power calculation is included in the Supporting Information. STATA 17.0 was used for the statistical analysis, and a statistical significance was considered with a *p* value ≤ 0.05.

## 3 Results

### 3.1 Patient Population

Twenty‐five participants were recruited, which was a higher number than originally planned due to a higher drop‐out rate due to either illness or unwillingness to undergo a second biopsy. Eighteen participants (8 females/10 males; median age: 25 [22; 35]; 16 nonsmokers, 2 smokers [ca. 5 cig/day]) underwent both biopsies and were included in the analysis; seven participants (4 females/3 males; median age: 23 [22; 24]) were considered as drop‐outs due to various reasons (Figure 4). The dropouts did not differ significantly from the finally analyzed population regarding gender distribution, age, or initial group allocation. Among the 18 analyzed ones, the thickness of the palatal tissue prior to biopsy harvesting showed no significant difference between the control (mean [SD]: 4.55 [1.4] mm) and test (mean [SD]: 4.74 [1.3] mm) site (*p* = 0.617).

**FIGURE 4:**
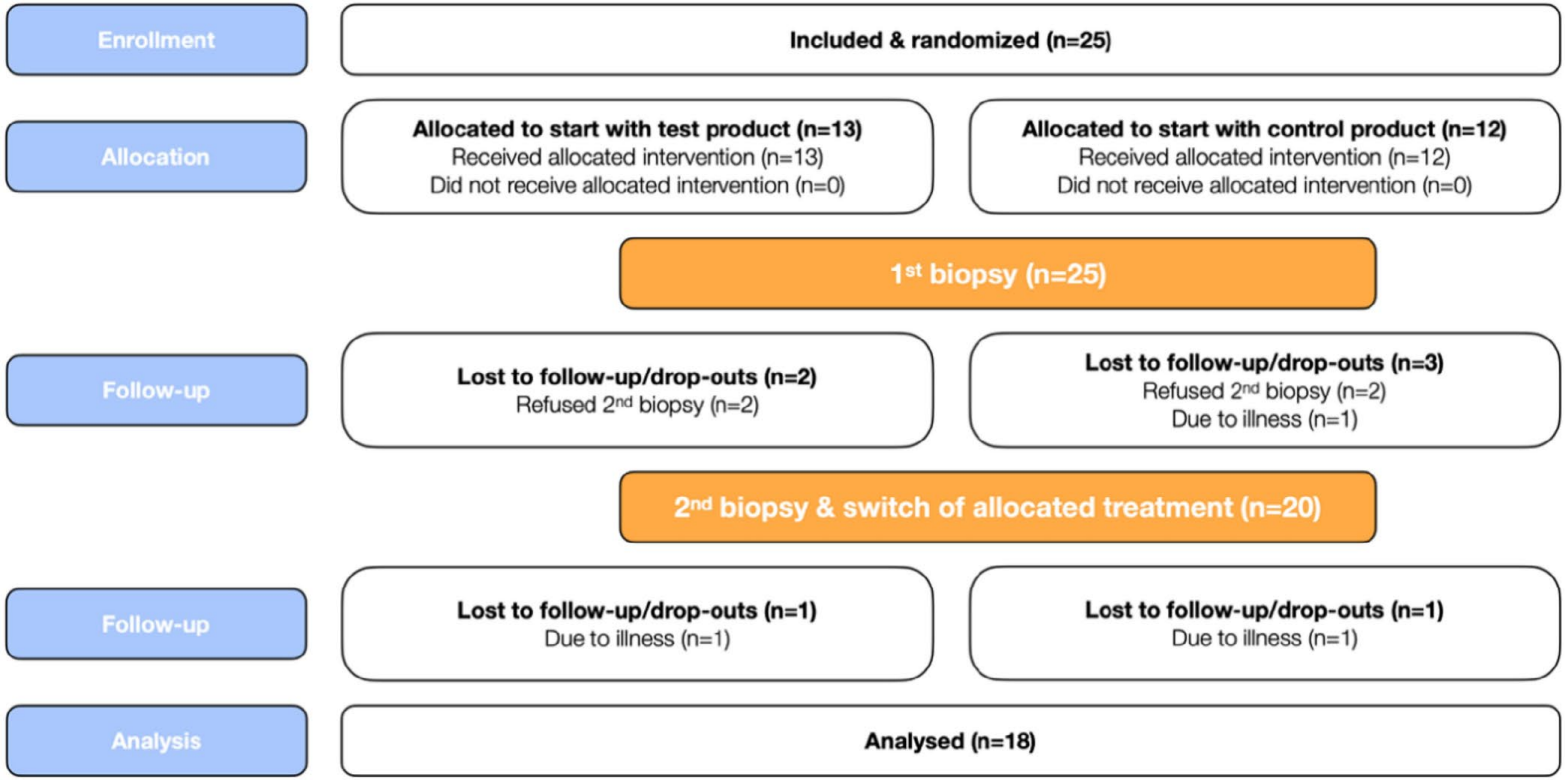
CONSORT flow diagram.

### 3.2 Primary Outcome Parameter—Pain Perception (VAS 0–10)

Independent of the allocated treatment, the participants reported pain perception up to 7 days after harvesting of the biopsy, except for two participants reporting minor pain perception even on Day 14. The highest values were reported on Day 1, followed by Days 2, 7, and 3; that is, participants often reported slightly increased pain levels on Day 7 compared to Day 3. In general, high interindividual variation was observed, but median VAS values stayed at each time point < 3.5 (Table 1, Figure 5). The mixed effect linear regression analysis did not show any significant effect of group allocation on the main outcome parameter pain perception (*p* = 0.275); neither did gender (*p* = 0.408), age (*p* = 0.373), and palatal thickness (*p* = 0.621). Only the parameter time showed an effect with a significantly reduced pain perception at all follow‐ups after Day 1 (*p* < 0.001) (Table 2).

**TABLE 1:** Summary of the assessed parameters at the various time points.

		Day 1	Day 2	Day 3	Day 7	Day 14	Day 21
Pain perception (VAS), median (Q1; Q3)	Control	3.3 (1.8; 4.9)	1.2 (0.4; 2.7)	0.8 (0; 1.2)	1.0 (0.2; 1.4)	0 (0; 0)	0 (0; 0)
	Test	2.3 (1.2; 5.1)	1.3 (0.5; 2.9)	0.8 (0; 2.3)	1.2 (0.6; 2.3)	0 (0; 0)	0 (0; 0)
Difficulties with eating/drinking (VAS), median (Q1; Q3)	Control	4.4 (1.6; 4.9)	1.6 (0.7; 2.9)	0.9 (0; 2.4)	1.3 (0; 3.1)	0 (0; 0)	0 (0; 0)
	Test	3.2 (1.9; 7.7)	2.4 (0.6; 4.1)	1.5 (1; 2.7)	1.8 (0.5; 2.9)	0 (0; 0)	0 (0; 0)
Limitations reg. food consumption, yes; *n* (%)	Control	12 (66.7)	10 (55.6)	6 (33.3)	12 (66.7)	1 (5.6)	0 (0)
	Test	14 (77.8)	13 (72.2)	12 (66.7)	12 (66.7)	1 (5.6)	0 (0)
Taste alterations (VAS), median (Q1; Q3)	Control	0.1 (0; 1.1)	0.1 (0; 0.5)	0 (0; 0.1)	0 (0; 0)	0 (0; 0)	0 (0; 0)
	Test	0 (0; 2.2)	0 (0; 0.4)	0 (0; 0)	0 (0; 0)	0 (0; 0)	0 (0; 0)
Area (mm^2^), mean (SD)	Control				22.1 (3.6)		
	Test				19.2 (2.9)^a^		
Volume deficiency (mm^3^), mean (SD)	Control				10.5 (9.8)	14.2 (7.4)^a^	7.8 (7.4)
	Test				8.0 (12.1)^a^	11.2 (8.0)	5.9 (8.2)^a^
Maximum defect depth (mm), mean (SD)	Control				0.84 (0.3)	0.88 (0.4)^a^	0.66 (0.3)
	Test				0.73 (0.4)^a^	0.75 (0.3)	0.64 (0.6)^a^
Color match (%), mean (SD)	Control			66.7 (12.1)^a^	72.6 (5.0)	82.3 (7.2)^a^	85.1 (6.6)
	Test			64.7 (11.5)	75.4 (8.3)^a^	82.5 (6.0)	83.9 (5.2)^a^

Abbreviations: Q1/3, first/third quartile; SD, standard deviation; VAS, visual analogue scale.

^a^ One missing value.

**FIGURE 5:**
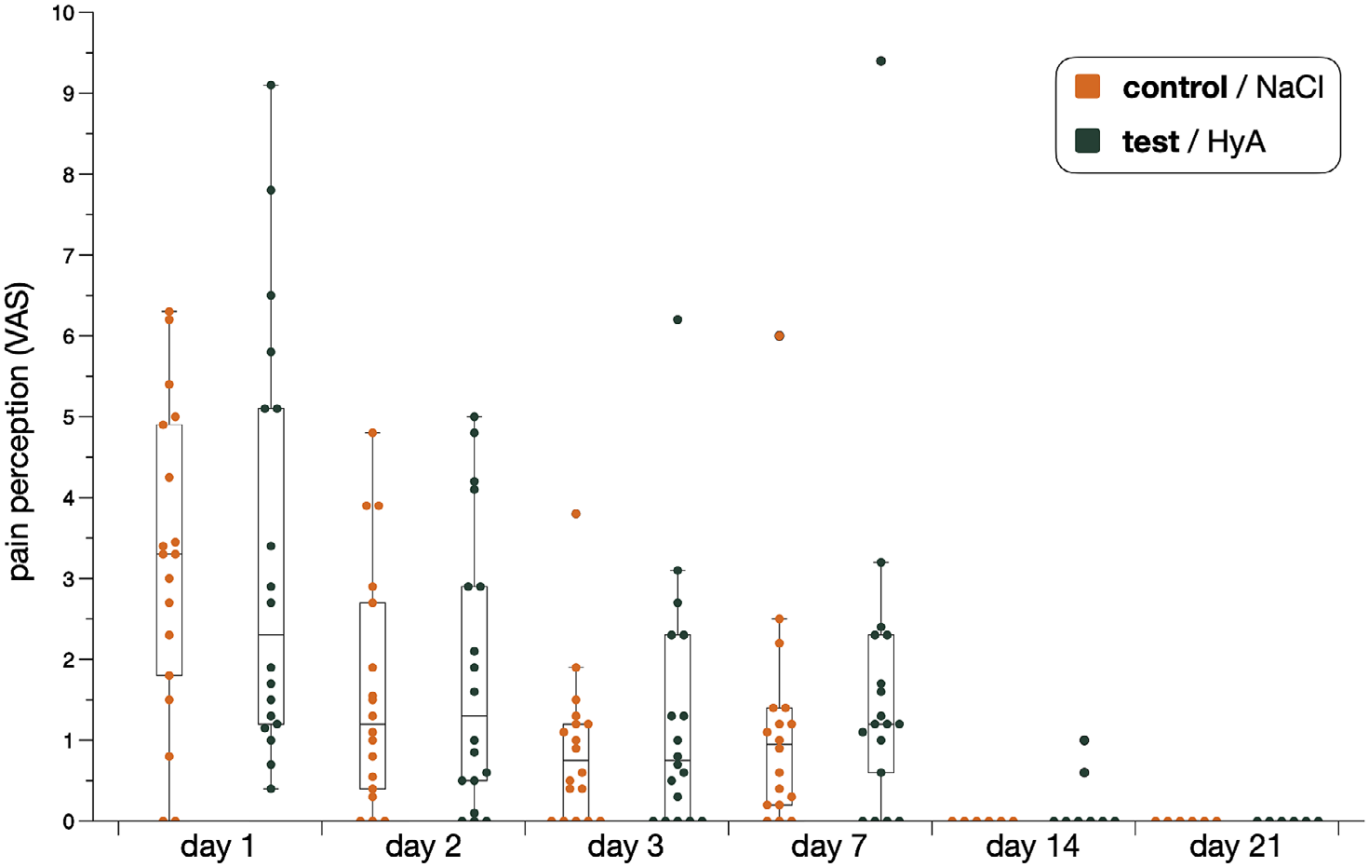
Pain perception (VAS) at Days 1, 2, 3, 7, 14, and 21.

**TABLE 2:** Multilevel mixed‐effects linear regression analysis with pain perception (VAS) as primary outcome parameter and group allocation as main predictor; a negative coefficient indicates a lower pain perception.

Parameter		Coef.	95% CI		*p*
			Lower	Upper	
Group allocation	Control/NaCl	Ref.			
	Test/HyA	0.24	−0.19	0.67	0.275
Gender	Male	Ref.			
	Female	−0.29	−0.99	0.40	0.408
Age	Years	−0.02	−0.06	0.02	0.373
Palatal thickness	mm	−0.05	−0.27	0.16	0.621
Time point	Day 1	Ref.			
	Day 2	−1.53	−2.10	−0.97	**< 0.001**
	Day 3	−2.17	−2.73	−1.60	**< 0.001**
	Day 7	−1.83	−2.39	−1.26	**< 0.001**
	Day 14	−3.20	−3.77	−2.64	**< 0.001**
	Day 21	−3.25	−3.81	−2.68	**< 0.001**
*R* ^2^ (conditional)	0.55				
*p* value	**< 0.001**				

*Note:* Bold values indicate statistical significance.

Abbreviations: CI, confidence interval; Coef., coefficient; HyA, hyaluronic acid; Ref., reference group.

### 3.3 Secondary Outcome Parameters—Additional Patient‐Reported Outcomes (PRO)

Participants took in total for the control and test site 0.5 (0; 3) and 1 (0; 3) pain killers [median (Q1; Q3)], respectively; a maximum of 14 and 4 tablets was reported for a control and test site, respectively. Group allocation had no significant effect on the total amount of consumed pain killers but indicated a weak tendency for fewer pain killers at the test site (*p* = 0.115) (Table S1). Difficulties with eating/drinking (VAS 0–10) showed a similar behavior/curve as pain perception and up to Day 7 about 2/3 of the participants reported independent of the group allocation to experience limitations regarding food consumption. The median VAS values stayed at each time point < 4.5 (Table 1) and group allocation had no significant effect on the extent of difficulties with eating/drinking (*p* = 0.098) but the difficulties with eating/drinking were significantly reduced over time (*p* ≤ 0.001) (Table S2). Taste alterations were in general seldomly reported (Table 1) and not affected by group allocation (*p* = 0.612) but significantly improved over time (*p* ≤ 0.021) (Table S2). Postoperative bleeding occurred for participants at the control and test site in total 1 (0; 2) and 1 (0; 4) time [median (Q1; Q3)] with almost all of them (i.e., 87.8% of all bleeding events) occurring within the first day; a maximum of 7 and 10 events was reported for a control and test site, respectively. Group allocation had no significant effect on the total number of bleeding events (*p* = 0.419) (Table S1). Finally, 8 (44.4%) participants reported an overall positive experience after applying the test product, while there was only one participant reporting this for the control product; this represented a significant difference in favor of the test group (Exact McNemar: *p* = 0.039).

### 3.4 Secondary Outcome Parameters—Area and Volumetric Measurements

The area of the remaining open wound was assessed on Day 7 and the mean value was about 3 mm^2^ smaller at the test compared to the control sites (Table 1). The mixed effect linear regression analysis showed a significant effect of group allocation favoring the test product, that is, the application of the test product resulted in a significantly smaller wound area after 7 days (*p* < 0.001). In addition, a tendency was detected, that a thicker palatal tissue was associated with a faster wound healing on Day 7 (*p* = 0.069) (Table S1). Both the volume deficiency and maximum defect depth showed at all time points slightly smaller values for the test sites (i.e., indicating a smaller remaining defect/faster wound healing) (Table 1), but group allocation lacked statistical significance in the mixed effect linear regression analyses (volume deficiency: *p* = 0.084; maximum defect depth: *p* = 0.117). Interestingly, the volume deficiency was at Day 14 significantly bigger compared to Day 7 (*p* = 0.002). Finally, the maximum defect depth was at Day 21 significantly reduced compared to Day 7 (*p* = 0.039) (Table S2).

### 3.5 Secondary Outcome Parameters—Color Matching and Visual Judgment of Epithelization and Wound Visibility

The color match to the surrounding tissue increased independently of group allocation from Day 3 until 21 but did not reach 100% within the study duration (Table 1, Figure 6). Group allocation had no significant effect on the color match (*p* = 0.986) but color match significantly improved over time (*p* ≤ 0.001) (Table S2). The harvesting site was for all participants consistently judged to be still visible after 21 days. Regarding re‐epithelization, in about 10% of all photographs the judgment/scoring was made after group discussion; re‐epithelization was earliest visible after 14 days (in 50% of the test and control sites) and was complete in 66.7% and 77.8% of the control and test sites, respectively, after 21 days.

**FIGURE 6:**
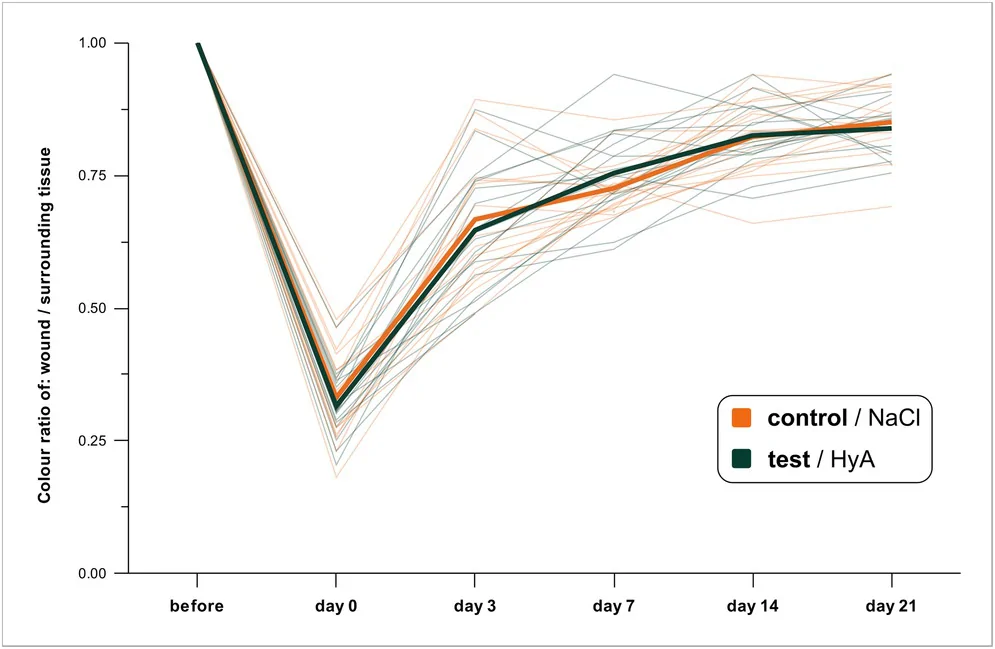
Color match to the surrounding tissue; opaque lines represent the averages per group, and transparent lines represent each individual site.

## 4 Discussion

The present RCT evaluated the effect of repeated local application of a HyA containing gel on patient morbidity and wound healing after a palatal punch‐biopsy representing a FGG. While wound closure rate was significantly improved by repeated local application of a HyA containing gel, pain perception and discomfort were not statistically significantly different compared to the control group. An overall positive experience was reported when applying the HyA containing gel.

Products containing HyA have indeed been tested previously to promote palatal wound healing (Joshi et al. 2024) as well as healing in other indications in the oral cavity, for example, after oral biopsy harvesting (Romeo et al. 2014), tooth extraction (Domic et al. 2023), bone regeneration procedures (Bayyigit et al. 2026), or for the management of recurrent aphthous stomatitis (Al‐Maweri et al. 2021), oral lichen planus (Waingade et al. 2022), and recurrent oral ulcers in Behçet's disease patients (Lee et al. 2008). This is based on the overall multiple positive effects of HyA on wound healing, such as promoting blood clot formation and stability, collagen maturation, cell proliferation, and angiogenesis (Weigel et al. 1986; Tolg et al. 2014; Asparuhova et al. 2019; Nyman et al. 2019; Taskan et al. 2021; Pilloni et al. 2023, 2025), as well as on reducing pain perception by downregulating prostaglandin E2 and cyclooxygenase 2 related inflammatory pathways (Yasuda 2010). In addition to pain perception, various parameters have been recorded herein, that is, wound area, volume deficiency, maximum defect depth, and color match, to objectively judge the progress of palatal wound healing. Indeed, the repeated local application of a HyA containing gel led to a significantly faster re‐epithelialization of the palatal wound, that is, the remaining wound area was after 7 days in the test group about 3 mm^2^ smaller compared to the control group. As the area of a 6‐mm‐diameter circle is about 28 mm^2^, this difference corresponds to approximately 10% of the original wound area, and expressed in percentage, the wound size reduction after 7 days was 32.2% and 21.9% in the test and control group, respectively. These findings agree well with previous preclinical (Hammad et al. 2011) and clinical studies (Yıldırım et al. 2018; Alpan and Cin 2023) showing accelerated re‐epithelization after HyA application in palatal wounds. Interestingly, in a similar study set up, different collagen‐based wound dressings lead to a comparable wound size reduction of 30%–40% after 7 days (Bienz et al. 2024). However, the control group in this specific study achieved a 50% wound size reduction, thus exceeding significantly within the same timeframe the results of the control group herein; nevertheless a direct comparison between the studies might be difficult due to differences in measurement set ups, differences in postoperative care, that is, usage of a palatal splint, etc. (Bienz et al. 2024).

Other objective outcome parameters assessed herein, such as volume deficiency and maximum defect depth, indicated a similar trend, but lacked statistical significance. Interestingly, the volume deficiency was in both groups at Day 14 significantly larger compared to Day 7 and Day 21. This paradox exemplifies the challenges experienced herein with analyzing/interpreting data from intraoral scanning. Intraoral scanning is a tempting method and has received wide‐spread popularity (Tavelli, Barootchi, Majzoub, et al. 2021), but it comes along with some challenges, especially for the early postoperative period, that must be mentioned. Defect scanning immediately after graft harvesting is likely influenced by tissue “swelling” due to local anesthesia and a scanning during the first postoperative days by the tissue swelling due to the invasive procedure itself introducing a relevant risk for bias when assessing defect depth. In addition, even if the potential bias can be minimized by selecting stable anatomical landmarks (i.e., teeth and a palatal area unaffected by any swelling) for the alignment of consecutive scans, it should be kept in mind that any swelling adjacent to the defect area fosters tissue elevation and thereby any defect measurements are distorted and do not represent the true defect depth. Indeed, previous studies using intraoral scanning failed to show the actual defect depth, that is, although a 3 mm thick biopsy was taken the mean distance compared to the preoperative scan never exceeded 0.5 mm (Bienz et al. 2024). Another factor, influencing accuracy of defect depth registration by intraoral scanning can be the presence of excessive coagulate and/or granulation tissue. Thus, it was decided herein not to analyze the scanning from 3 days post intervention. In addition, and irrespective of the impact of tissue alterations, it should be acknowledged that stitching errors (Revilla‐León et al. 2025) may occur during the IOS scanning procedure impairing a “perfect” alignment of the scans. Furthermore, the best‐fit alignment algorithm strives to evenly minimize mesh distances between respective surface points and thus averages positive/negative deviations regardless of the clinical result (O'Toole et al. 2019).

Herein, several subjective outcome parameters, that is, PRO, were also assessed, such as pain perception, need of pain killer consumption, difficulties/limitations with food consumption, and experience of any taste alteration. The tested HyA product was rated overall as easy‐to‐apply measure, providing in about half of the patients a positive experience after application, which was statistically superior to the control group; however, the statistical test used does not consider potential confounders, such as palatal thickness. This might explain why all other PRO, which were tested in multilevel mixed‐effects linear regression analyses, did not show any significant difference between the HyA and control group using a placebo. Furthermore, any instant or fleeting perception of a positive experience, for example, due to a more pleasant taste of the gel—described as apple and pear by the producer—compared to the salty physiological saline, may not necessarily impact on the overall pain perception or on any of the PRO. In this context, the discrepancy that a faster wound healing did not reduce patient morbidity might be due to the limited wound size herein and agrees with previous reports. For example, in a study assessing the effect of HyA on wound healing after oral biopsies of similar dimension (i.e., about 30 mm^2^), pain perception was not reduced despite a faster wound healing after 7 days (Romeo et al. 2014). In contrast, other studies with a bigger wound size (i.e., a FGG of 10–15 × 5–8 mm) reported enhanced/faster wound healing and less pain perception after application of a HyA containing product (Yıldırım et al. 2018; Alpan and Cin 2023).

Disparities/discrepancies between results of objective and subjective outcome parameters have been previously reported in similar studies on palatal wound healing, either within the same study or among studies. This fact and the overall low number of studies with direct comparisons of different products/approaches may explain why no gold standard for the treatment of palatal wounds has been identified so far (Silva et al. 2023; de Almeida et al. 2024; Jankowski et al. 2024). In this context, using a soft tissue substitute product—and thus avoiding harvesting a soft tissue graft—would largely minimize postoperative morbidity; however, in both main indications, that is, to increase the width of keratinized tissue or the thickness of gingival/mucosal tissue, currently available soft tissue substitutes are inferior to autogenous grafting, which remains the gold standard (Bertl, Gotfredsen, et al. 2017; Bertl, Melchard, et al. 2017; Thoma et al. 2018; Bertl et al. 2021; Montero et al. 2022; Valles et al. 2022; Rotundo et al. 2024). This in turn makes assessment of products/approaches promoting palatal wound healing and reducing postoperative morbidity highly interesting. The present study overcomes several limitations of previous studies on this topic. More specifically, (1) the graft had a standardized dimension, (2) there was no bias due to a second surgical site, possibly interfering with patients' pain perception and morbidity (especially relevant if the recipient site was in the upper jaw), (3) there was no bias from any additional measures needed for the postoperative care of the recipient site (e.g., chlorhexidine rinsing, use of antibiotics, etc. as in studies involving graft harvesting for a clinical indication), and (4) there was no variation due to the often high interindividual pain perception (Burkhardt et al. 2015) as the study had a crossover design. In this context, as the test and control products were locally applied for only 7 days, any carryover effect on the other side of the palate should not be expected or it would be extremely minimal, and if any, it should have been washed‐out after the additional 2 weeks of no use; therefore “period” was also not included in the regression analysis. Nevertheless, the possibility of a carryover effect, in terms of that the patients were sensitized from the first procedure and experienced either more or less pain during the second procedure due to knowing what to expect, could not be excluded. Therefore, group allocation was randomized, that is, half of the patients started with the test product, while the other half started with the control, a measure that should have balanced any possible carryover effect and that led to the decision of not including “sequence” in the regression analysis. However, the study also has some limitation; due to ethical reasons the size of the wound was relatively small (6 mm in diameter), and this likely contributed to the lack of difference in pain perception, despite the faster wound healing. In addition, this set‐up somehow limits the external validity of the present study, as in the clinic the graft dimension is, in most cases, exceeding a circle of 6 mm. Finally, the test and control agent had different consistencies, that is, use of physiological saline solution as control was not optimal, and a gel with similar consistency and characteristics as the test gel, but without HyA, would have been more appropriate, but could not be provided. However, both product packages were masked/unidentified, treatment allocation was randomized, and the participants were blinded regarding treatment group allocation and not explicitly informed on the consistency (gel or fluid) of the test or control. Nevertheless, the chance that the participants recognized which product was the test and which the control cannot be excluded and this introduces a potentially high risk of bias regarding the PRO. Side effects of the HyA containing gel have not been noticed herein and are in general seldomly reported in the literature; if side effects have been previously reported after intraoral HyA application, they were related to injections of HyA into the tissues (Bertl, Gotfredsen, et al. 2017; Vaquero Martinez et al. 2023).

Another relevant point that should be discussed is the dose and chemical form of the HyA containing product and the application mode used in this study. It cannot be excluded that the 0.3%, non‐crosslinked, middle molecular weight HyA used herein had a too low dose and/or remained in the wound for a too short time—despite repeated applications—to exert a relevant pharmacological action for a perceivable effect. Repeated local applications were chosen herein with the rationale that the wound created in the palate is basically nonself‐contained and the wound area is directly exposed to the saliva and tongue, and thus reasonably and much likely any applied product would be largely removed within a very short timeframe. For example, a previous study (Yıldırım et al. 2018) comparing different concentrations of a non‐crosslinked, high molecular weight HyA gel (i.e., 0.2% and 0.8% HyA) under a surgical paste, showed improved wound healing in the HyA treated groups compared to the control. Thus, more research is needed to define the optimal dose, crosslinking extent, and mode of application of HyA containing products in this indication (Joshi et al. 2024).

In this context, two interesting aspects might be the production of mucoadhesives/films with HyA, which are supposed to remain longer on the palatal wound (Nabiyi et al. 2024; Lee et al. 2024), and the combination with a stent/splint and/or periodontal dressing. One study (Lee et al. 2024) comparing a HyA gel with a HyA film in a preclinical as well as in an ex vivo model reported overall better results for the film. The alternative to use a pre‐ or chair‐side‐fabricated stent aims also to keep the product longer in place. However, as a stent as such also reduces overall pain perception (Silva et al. 2023; Basma et al. 2023; Alkaya et al. 2023), it remains unclear whether the combination of a stent with a HyA containing product would provide accumulative benefits. Some indications can be drawn from previous studies, where for example HyA was applied with a stent compared to a periodontal dressing (Baroudi and Othman 2024) or HyA with a collagen sponge and periodontal dressing compared to the same combination without HyA (Khalil et al. 2022) resulted in less pain; further, HyA with a gelatine sponge compared to a gelatine sponge only improved the rate of re‐epithelialization (Parlak et al. 2023).

In conclusion and within the limitations of this RCT, such as the possibility of a carryover effect and questionable blinding of the participants, the results suggest that repeated local application of a 0.3% HyA containing gel appears to accelerate wound healing in terms of re‐epithelialization in an open palatal wound, but other parameters, such as volume deficiency and maximum defect depth, did not differ significantly between the groups. Furthermore, the HyA containing gel was considered an easy‐to‐apply measure and provided in about 1 out of 2 patients a positive experience after application but did not improve any PRO in such small‐sized wounds compared to sterile saline application.

## Author Contributions

**Madeleine Dahi:** project administration, data curation, writing – original draft, investigation. **Sara Amash:** data curation, project administration, writing – original draft, investigation. **Kristina Bertl:** conceptualization, writing – review and editing, project administration, data curation, formal analysis, funding acquisition, investigation. **Florian Beck:** data curation, formal analysis, writing – review and editing. **Patrick Heimel:** data curation, formal analysis, writing – review and editing. **Andreas Stavropoulos:** conceptualization, writing – original draft, resources, supervision, funding acquisition, project administration.

## Funding

Funding for this study was obtained from the Austrian Society for Implantology (ÖGI). The HyA product (GUM Aftaclear Gel) was provided free of charge by Sunstar (Europe SA, Etoy, Switzerland). No other external funding was acquired.

## Ethics Statement

The study protocol was approved by the regional ethical review board (Dnr. 2020‐01018) and all patients provided written consent prior to inclusion.

## Conflicts of Interest

Andreas Stavropoulos is a member of the editorial board of the COIR; otherwise, all authors declare no conflicts of interest.

## Supporting information


**Data S1:** CONSORT checklist of information to include when reporting randomized crossover trial.

**Table S1:** Multilevel mixed‐effects linear regression analysis with (1) pain killer consumption (total amount), (2) bleeding events (total number), and (3) defect area at Day 7 as primary outcome parameter and group allocation as main predictor; a negative coefficient indicates (1) consumption of fewer pain killers, (2) fewer bleeding events, and (3) smaller defect area. As these parameters were measured only once, the parameters “time point” and “Interaction time point with group allocation” were not included.
**Table S2:** Multilevel mixed‐effects linear regression analysis with (1) difficulties with eating/drinking (VAS), (2) taste alterations (VAS), (3) volumetric deficiency, (4) maximum defect depth, and (5) color matching as primary outcome parameters and group allocation as main predictor; a negative coefficient indicates (1) fewer difficulties with eating/drinking, (2) fewer taste alterations, (3) reduced volumetric deficiency, (4) smaller defect depth, and (5) inferior color matching.

## Data Availability

The data that support the findings of this study are available from the corresponding author upon reasonable request.
