# Supplementary material for: Effect of Hyaluronic Acid on Palatal Wound Healing—A Randomized Crossover Clinical Trial
Source: Clin Oral Implants Res. 2026 Jul 16;37(10):1184–96. doi: 10.1111/clr.70159 (PMC13644540; doi:10.1111/clr.70159)
Supplement: Supplementary file 2 [file CLR-37-1184-s002.docx]

**Supplementary methods.** Rational for the sample size calculation.

Yildrim et al. (2018) was judged as relevant study for the sample size calculation. Yildrim et al. reported at day 3 a mean difference of the 2 HyA groups compared to the control group of 45 to 47.5mm (with a calculated SD of mean difference of 24 to 26mm) and at day 7 a mean difference of 32.5 to 36.7mm (with a calculated SD of mean difference of about 27mm). Taking these values from day 3 and 7 would have resulted in the following sample size:

- 90% power, 45mm mean difference, SD 25mm: 3.2 participants
- 90% power, 35mm mean difference, SD 30mm: 7.7 participants

Due to this low sample size, we included the information provided in Olsen et al. (2017), who reported, that a minimum clinically important difference in acute pain values ranged widely from 8 to 40 mm. Based on these values, it was decided that to show at least a difference of 25mm with a conservative SD of 30mm, which resulted in 15.1 participants, which was rounded to 16.

**Supplementary table 1.** Multilevel mixed-effects linear regression analysis with 1) pain killer consumption (total amount), 2) bleeding events (total number), and 3) defect area at day 7 as primary outcome parameter and group allocation as main predictor; a negative coefficient indicates 1) consumption of fewer pain killers, 2) fewer bleeding events, and 3) smaller defect area. As these parameters were measured only once, the parameters “timepoint” and “Interaction time point with group allocation” were not included.

|  | | **1) Pain killer consumption (total amount)** | | | | **2) Bleeding events (total number)** | | | | **3) Area at day 7 (mm^2^)** | | | |
| --- | --- | --- | --- | --- | --- | --- | --- | --- | --- | --- | --- | --- | --- |
| **Parameter** | | **Coef.** | **95% CI** | | **p-value** | **Coef.** | **95% CI** | | **p-value** | **Coef.** | **95% CI** | | **p-value** |
|  |  |  | ***Lower*** | ***Upper*** |  |  | ***Lower*** | ***Upper*** |  |  | ***Lower*** | ***Upper*** |  |
| **Group allocation** | *Control/NaCl* | Ref. |  |  |  | Ref. |  |  |  | Ref. |  |  |  |
|  | *Test/HyA* | -1.21 | -2.71 | 0.30 | 0.115 | 0.40 | -0.58 | 1.39 | 0.419 | -2.79 | -3.92 | -1.65 | **< 0.001** |
| **Gender** | *Male* | Ref. |  |  |  | Ref. |  |  |  | Ref. |  |  |  |
|  | *Female* | 0.67 | -1.71 | 3.05 | 0.583 | -0.68 | -2.29 | 0.94 | 0.414 | 0.59 | -2.17 | 3.35 | 0.675 |
| **Age** | *Years* | -0.35 | -0.17 | 0.10 | 0.615 | -0.07 | -0.17 | 0.02 | 0.117 | 0.02 | -0.14 | 0.18 | 0.795 |
| **Palatal thickness** | *mm* | 0.22 | -0.51 | 0.95 | 0.557 | -0.08 | -0.57 | 0.41 | 0.747 | -0.58 | -1.20 | 0.05 | 0.069 |
| **R^2^** (conditional) | | 0.92 | | | | 0.93 | | | | 0.96 | | | |
| **p-value** | | 0.503 | | | | 0.475 | | | | **< 0.001** | | | |

*CI, confidence interval; Coef., coefficient; HyA, hyaluronic acid; Ref., reference group.*

*Bold values indicate statistical significance.*

**Supplementary table 2.** Multilevel mixed-effects linear regression analysis with 1) difficulties with eating/drinking (VAS), 2) taste alterations (VAS), 3) volumetric deficiency, 4) maximum defect depth, and 5) color matching as primary outcome parameters and group allocation as main predictor; a negative coefficient indicates 1) fewer difficulties with eating/drinking, 2) fewer taste alterations, 3) reduced volumetric deficiency, 4) smaller defect depth, and 5) inferior color matching.

|  | | **1) Difficulties with eating/drinking (VAS)** | | | | **2) Taste alterations (VAS)** | | | | **3) Volumetric deficiency** | | | | **4) Maximum defect depth** | | | | **5) Color matching** | | | |
| --- | --- | --- | --- | --- | --- | --- | --- | --- | --- | --- | --- | --- | --- | --- | --- | --- | --- | --- | --- | --- | --- |
| **Parameter** | | **Coef.** | **95% CI** | | **p-value** | **Coef.** | **95% CI** | | **p-value** | **Coef.** | **95% CI** | | **p-value** | **Coef.** | **95% CI** | | **p-value** | **Coef.** | **95% CI** | | **p-value** |
|  |  |  | ***Lower*** | ***Upper*** |  |  | ***Lower*** | ***Upper*** |  |  | ***Lower*** | ***Upper*** |  |  | ***Lower*** | ***Upper*** |  |  | ***Lower*** | ***Upper*** |  |
| **Group allocation** | *Control/NaCl* | Ref. |  |  |  | Ref. |  |  |  | Ref. |  |  |  | Ref. |  |  |  | Ref. |  |  |  |
|  | *Test/HyA* | 0.44 | -0.08 | 0.96 | 0.098 | 0.06 | -0.17 | 0.30 | 0.612 | -2.37 | -5.09 | 0.35 | 0.087 | -0.09 | -0.19 | 0.02 | 0.117 | -0.02 | -2.27 | 2.23 | 0.986 |
| **Gender** | *Male* | Ref. |  |  |  | Ref. |  |  |  | Ref. |  |  |  | Ref. |  |  |  | Ref. |  |  |  |
|  | *Female* | -0.33 | -1.11 | 0.46 | 0.419 | -0.10 | -0.72 | 0.51 | 0.747 | -0.03 | -6.62 | 6.55 | 0.993 | -0.12 | -0.38 | 0.14 | 0.358 | 0.18 | -4.26 | 4.61 | 0.938 |
| **Age** | *Years* | -0.42 | -0.09 | 0.00 | 0.064 | 0.00 | -0.03 | 0.04 | 0.879 | 0.22 | -0.16 | 0.59 | 0.253 | 0.01 | -0.01 | 0.03 | 0.188 | 0.12 | -0.13 | 0.37 | 0.338 |
| **Palatal thickness** | *mm* | -0.03 | -0.28 | 0.22 | 0.805 | -0.04 | -0.17 | 0.10 | 0.591 | 0.28 | -1.24 | 1.80 | 0.718 | 0.02 | -0.04 | 0.08 | 0.461 | 0.15 | -1.05 | 1.34 | 0.811 |
| **Timepoint** | *Day 1* | Ref. |  |  |  | Ref. |  |  |  |  | | | |  | | | |  | | | |
|  | *Day 2* | -1.72 | -2.39 | -1.04 | **< 0.001** | -0.48 | -0.88 | -0.07 | **0.021** |  |  |  |  |  |  |  |  |  |  |  |  |
|  | *Day 3* | -2.36 | -3.04 | -1.69 | **< 0.001** | -0.73 | -1.14 | -0.33 | **< 0.001** |  |  |  |  |  |  |  |  | Ref. |  |  |  |
|  | *Day 7* | -2.05 | -2.73 | -1.38 | **< 0.001** | -0.71 | -1.12 | -0.31 | **0.001** | Ref. |  |  |  | Ref. |  |  |  | 8.33 | 5.16 | 11.49 | **< 0.001** |
|  | *Day 14* | -3.95 | -4.63 | -3.27 | **< 0.001** | -0.98 | -1.39 | -0.58 | **< 0.001** | 3.35 | 1.25 | 5.46 | **0.002** | 0.03 | -0.10 | 0.15 | 0.685 | 16.74 | 13.58 | 19.90 | **< 0.001** |
|  | *Day 21* | -4.01 | -4.69 | -3.34 | **< 0.001** | -1.03 | -1.44 | -0.63 | **< 0.001** | -2.31 | -4.41 | -0.20 | **0.032** | -0.13 | -0.26 | -0.01 | **0.039** | 18.85 | 15.68 | 22.01 | **< 0.001** |
| **R^2^** (conditional) | | 0.56 | | | | 0.39 | | | | 0.76 | | | | 0.55 | | | | 0.62 | | | |
| **p-value** | | **< 0.001** | | | | **0.001** | | | | **< 0.001** | | | | 0.089 | | | | **< 0.001** | | | |

*CI, confidence interval; Coef., coefficient; HyA, hyaluronic acid; Ref., reference group; VAS, visual analogue scale.*

*Bold values indicate statistical significance.*

**Supplementary statistical analysis.** Post-hoc power calculation.

A power calculation for the main outcome parameter (pain perception) was performed and indicated a power of 67.7%.
